# Hypoxia-inducible factor expression is related to apoptosis and cartilage degradation in temporomandibular joint osteoarthritis

**DOI:** 10.1186/s12891-022-05544-x

**Published:** 2022-06-16

**Authors:** Jun Zhang, Yu Hu, Zihan Wang, Xuelian Wu, Chun Yang, Hefeng Yang

**Affiliations:** 1 Yunnan Key Laboratory of Stomatology, School of Stomatology, Kunming Medical University, He Cheng Guo Ji Building C, 1088 Mid-Haiyuan Road, Kunming, 650100 Yunnan China; 2 Department of Orthodontics, Kunming Medical University Affiliated Stomatological Hospital, Kunming, China; 3 Honghe Health Vocational Collage, Honghe, Yunnan Province China

**Keywords:** Condyle, Osteoarthritis, Apoptosis, Cartilage degradation

## Abstract

**Background:**

It remains unclear etiology of cartilaginous tissues in osteoarthritis (OA) lesions. In this study, we hypothesized the accumulation of hypoxia-inducible factor (HIF) and activated apoptosis relate to condylar cartilage degeneration in vivo.

**Methods:**

Malocclusion stress was applied for 2 weeks, 4 weeks and 8 weeks to induce an OA-like lesion animal model in rats. Histological analysis was performed by H&E staining and Safranin O/fast green staining. The expression levels of protein in condylar cartilage were examined by immunostaining to evaluate cartilage degeneration.

**Results:**

We found apparent histological phenotypes associated with degeneration in the occlusion disorder (OD) stress group. The OD group at 4 weeks and 8 weeks had obviously reduced expression of Aggrecan (Acan) and type II collagen (Col II) in cartilage. In contrast, the OD groups had higher levels of ADAM metallopeptidase with thrombospondin type 5 (ADAMTS5) and matrix metallopeptidase 13 (MMP13) in the condylar cartilage than the control group. Moreover, the OD group cartilage had prominent degenerative changes with reduced levels of hypoxia inducible factor 1 alpha (HIF1α) and increased levels of hypoxia inducible factor 2 alpha (HIF2α) and the apoptosis factor Caspase3 in condylar cartilage at 8 weeks.

**Conclusion:**

Thus, abnormal hypoxic conditions inducing Occlusion disorder stress results in cartilage degeneration. opposite expression patterns of HIF1α and HIF2α could be involved in the pathogenesis of condylar cartilage degeneration and chondrocyte apoptosis. HIF2α may provide a potential negative feedback mechanism for HIF1α during cartilage damage.

**Supplementary Information:**

The online version contains supplementary material available at 10.1186/s12891-022-05544-x.

## Introduction

Temporomandibular joint (TMJ) is a synovial joint comprised of the mandibular condyle and glenoid fossa of the temporal bone [1]. TMJ osteoarthritis (TMJOA) is characterized by cartilage destruction and abnormal bone remodelling in subchondral bone [2]. However, the aetiology of TMJOA remains poorly elucidated. The TMJ has different morphological, functional, biomechanical and biological features compared with other joints in the body [3]. The most superficial cellular layer of the TMJ is fibrocartilage, which consists of type I collagen (Col I) and Col II [4]. Mandibular condylar cartilage acts as avascular connective tissue that functions autonomously to bear loads. Mechanical stress is associated with the pathogenesis condylar cartilage homeostasis disruption and the initiation of the catabolic pathway [5]. Cartilage degeneration occurs when abnormal mechanical stress continues to occur, which causes an imbalance in cartilage anabolism and catabolism [6]. Chondrocyte apoptosis and catabolic enzymes induce cartilage destruction to contribute to disease pathogenesis [7].

Condylar cartilage is a tissue that lacks blood vessels and nerves. Chondrocytes are located in a hypoxic or anoxic environment [8]. HIF1 and HIF2 appear to be the major regulators of the hypoxic response [9]. HIF1α exists only in hypoxic environments and exerts cytoprotective effects. HIF1α translocated to the nucleus and interacts with hypoxia-sensitive target genes to regulate angiogenesis, energy metabolism and cell proliferation and apoptosis under hypoxic conditions [10]. HIF2α is mainly expressed in highly differentiating chondrocytes and acts as a key catabolic transcription factor that mediates the hypertrophic differentiation of chondrocytes and cartilage degradation in osteoarthritic cartilage in humans and mice [11]. HIF2α is an essential catabolic factor in the pathophysiology of OA [12]. Moreover, HIF2α is involved in the initiation of blood vessel formation accompanied by increased vascular endothelial growth factor (VEGF) expression and the upregulation of multiple degradative enzymes, including MMP13 [13]. Blockade of HIF2α decreased cartilage degradation and related degradation factors [14]. When HIF2α was silenced, reactive oxygen species (ROS) and HIF1α expression was elevated in prehypertrophic cells [15].

Based on the anabolic role of HIF1α and the catabolic role of HIF2α, HIF1α and HIF2α have spatiotemporal expression differences during the pathologic process of OA [16]. However, the expression of HIF1α and HIF2α during the development of TMJOA is not well understood. Thus, we used OD rat models to show that the spatiotemporal expression pattern of HIF1α and HIF2α during OA development.

## Material and Methods

### Animals

All animal experiments complied with the Animal Research: Reporting of In Vivo Experiments (ARRIVE) guidelines and approved by Ethical Commitees of Kunming Medical University. Eight-week-old male Sprague–Dawley (SD) rats (weighing 160–180 g) were obtained from the Experimental Animal Department of Kunming Medical University and were randomly divided into the control (*n =* 10 rats) and OD groups (*n =* 30 rats). In the experimental group (OD group), disordered occlusion was created by abnormal dental occlusion force based on a previous report. Briefly, a ligation silk (0.25 mm diameter) knot was created on the first molar of maxillary to induce abnormal mechanical loading on the rat TMJ [16]. OD rats were sacrificed by dislocation of cervical vertebra after disordered occlusion for 2 weeks (*n =* 10 rats), 4 weeks (*n =* 10 rats), and 8 weeks (*n =* 10 rats).

### Histological staining

At the time of euthanasia, TMJ samples were dissected and fixed in 4% paraformaldehyde overnight. After be decalcified in 10% Ethylene Diamine Tetracetie Acid (EDTA) (pH 7.2–7.4), the samples were processed, embedded in paraffin and cut into 5 μm sections using a microtome (Leica, RM2235, Germany). Standard haematoxylin and eosin (H&E) staining was used to examine tissue histology. Safranin O and fast green staining were performed to determine proteoglycan changes, and the histological data were further analysed by assessing the Mankin scores [17] and Osteoarthritis research society international (OARSI) scores [18]. Semiquantitative Mankin scores and OARSI scores were significantly correlated and positively associated with modeling time. Without know the groups, three people grade scores according to Mankin scores and OARSI scores guideline with Safranin O staining slices. Each group has 10 rats, each rat right condylar had 6–8 Safranin O staining sections.

### Immunohistochemistry

Immunohistochemical analyses of sections of each construct were performed using an anti-rat HRP-DAB cell & tissue staining kit (R&D Systems, CTS017, USA). Sections were subjected to epitope recovery in citrate buffer at 99 °C for 30 min. Once the samples reached room temperature, the slides were washed in triethanolamine-buffered saline, and nonspecific immunoglobulin binding was blocked with 5% (V/V) bovine serum albumin for 30 min at room temperature. The sections were incubated overnight at 4 °C with the primary antibodies against Aggrecan (Acan, sc-166951,Santa Cruz Biotechnology, 1:100, USA); type collagen II (Col II, ab34712, Abcam, 1:100, UK); ADAM metallopeptidase with thrombospondin type 5 (ADAMTS5, ab182795, Abcam, 1:100), MMP13 (ab219620, Abcam, 1:100, UK), Caspase3 (sc-271759, Santa Cruz Biotechnology, 1:100, USA), Bcl-2 (B-cell lymphoma-2, sc-70411, Santa Cruz Biotechnology, 1:100, USA), HIF1α, (PA3-16,521, Thermo Fisher, 1:100, USA) and HIF2α (ab109616, Abcam, 1:100, UK). Saining specificity was confirmed by utilizing an isotype-matched immunoglobulin control (ab125938, Abcam, UK). All sections were incubated with a biotinylated secondary antibody, stained using an R&D HRP-DAB Staining Kit and counterstained with haematoxylin. After being mounted, the slides were photographed with microscope (Olympus, BX53, Japan). The numbers of positive cells in the cartilage layer were determined by ImageJ (National Institutes of Health). ImgeJ analysis percentages of brown positive cells in middle of condylar cartilage. Each group has 10 slices from different rats. All sections were placed on one slide and processed together under the same conditions.

### TdT-mediated dUTP Nick-End Labeling (TUNEL) assay

Multiple sections were stained using the In Situ Cell Death Detection Kit, AP (Roche Diagnostics Corp, Indianapolis, IN). Proteinase K concentration was varied from 25 μg/mL in phosphate buffered saline (PBS) and incubated from 20 min at 37° C. Tdt concentration was the recommended dilution. Anti-fluorescein antibody AP secondary substrate was incubated at 37° C.

### Statistical analysis

Comparisons between groups were evaluated with one-way analysis of variance (ANOVA) followed by Tukey’s test for multiple comparisons using SPSS 16.0 software (IBM, Armonk, NY, USA). Values of *P < 0.05, **P < 0.01, ***P < 0.005, and ****P < 0.001 were considered to indicate a significant difference between groups.

## Results

### Condylar cartilage degeneration in the OD rat models

**Fig. 1:**
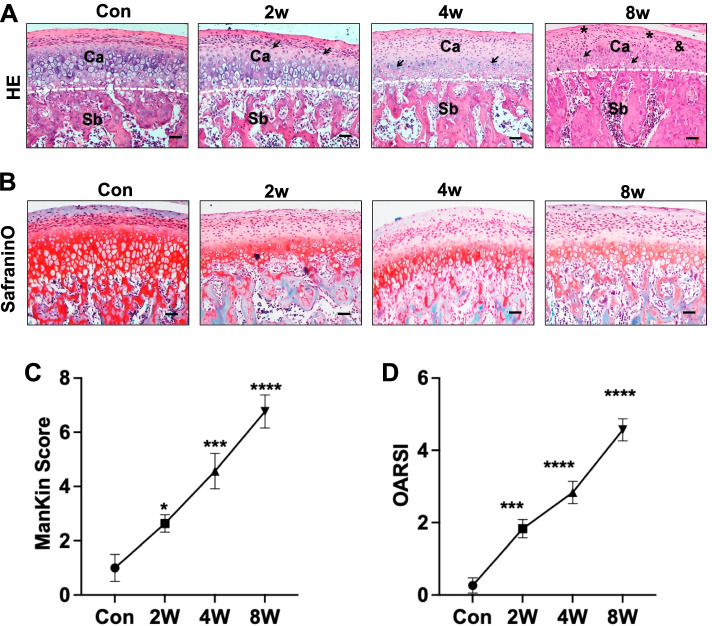
Condylar cartilage degeneration in rat models. Representative images of the first molar occlusion relationship in control and OD rats. H&E staining **A** (Ca: cartilage; Sb: subchondral bone; small superficial clusters is indicted by an “** → **”; fissures is indicated by an “*”) and Safranin O and fast green **B** analyses of glycosaminoglycans (red) in sagittal sections of the TMJ mandibular condylar cartilage layers. Scale bars: 50 μm. **C** Mankin scores and OARSI scores **D** in the control and OD groups. *P < 0.05, ***P < 0.005, ****P < 0.001. *N =* 6–8

H&E staining indicated that the mandibular condylar cartilage was divided into a fibrocartilage layer and subchondral bone. Compared with that of the controls, condylar cartilage with abnormal dental occlusion had structural and degradation changes. The 2-week, 4-week and 8-week OD groups had irregular surfaces. Starting at 2 weeks, the OD group had small superficial clusters. The 8-week OD group included fissures into the radial layer and had slight disorganization in the superficial cartilage layer (Fig. 1A). Safranin O fast staining showed that the distribution of proteoglycans in the controls was even and rich, whereas the OD group exhibited time-dependent cartilage degradation accompanied by extensive loss of proteoglycan areas and the total number of chondrocytes (Fig. 1B). Mankin scores based on H&E staining and safranin O staining were increased in the OD groups. In the OD groups, the Mankin scores (Fig. 1C) and OARSI scores (Fig. 1D) increased with time. These histomorphological staining results confirmed that the degeneration of condylar cartilage became serious with the extension of animal modelling time.

### Lower expression of condylar cartilage matrix protein in the OD rat models

**Fig. 2:**
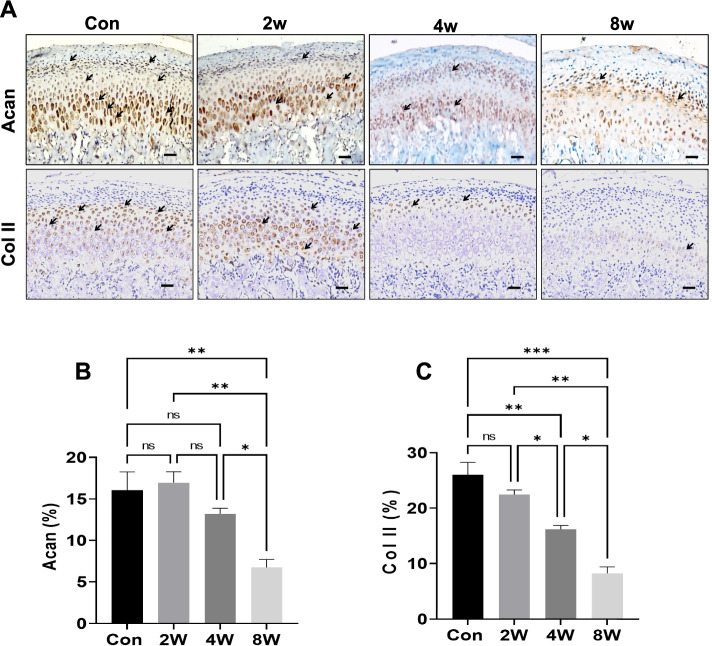
The expression of matrix proteins in condylar cartilage. Immunohistochemical analysis of Acan, Col II in mandibular condylar cartilage (**A**). Positive cells is indicted by an “** → **”.Scale bars 50 μm. Acan- (**B**), Col II- (**C**) positive (**D**) cells were counted in the cartilage layer. *P < 0.05, **P < 0.01. ***P < 0.005. *N =* 6

The expression of cartilage matrix proteins in the TMJ was assessed by immunohistochemistry (Fig. 2A). In the 2-week and 4-week OD groups, the level of Acan in condylar cartilage was close to that in the control group. At 8 weeks, the expression of Acan in the OD group was lower than that in the control group and the 2-week and 4-week OD groups (Fig. 2B). These results indicated that occlusion disorder for 8 weeks induced a decrease in Acan expression in the cartilage layer. The trend in the expression of Col II in cartilage was similar to that of Acan (Fig. 2C). Consequently, TMJ cartilage expression of synthesis proteins decreased in the OD group.

### Increased expression of extracellular matrix (ECM) degradation proteins in OD rat models

**Fig. 3:**
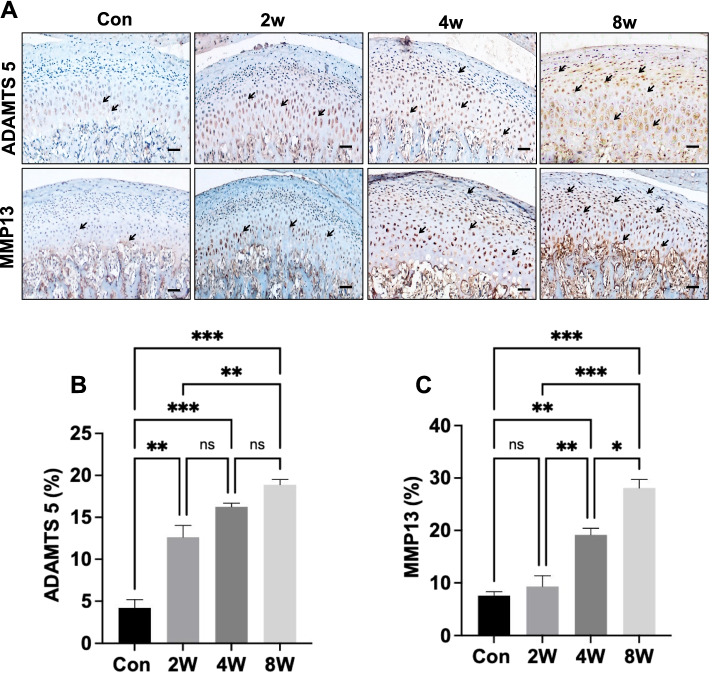
The expression of matrix degeneration-related proteins in the OD rat model. Immunohistochemical analysis of ADAMTS5 and MMP13 in mandibular condylar cartilage (**A**). Positive cells is indicted by an “** → **”. Scale bars: 50 μm. ADAMTS5- (**B**) and MMP13-positive (**C**) cells were counted in the cartilage layer. *P < 0.05, **P < 0.01, ***P < 0.005. *N =* 8

The protein expression of MMP13 and ADAMTS5 was lower in the control group than the OD groups. The expression of MMP13 began to increase at 2 weeks and continued to increase in the 8-week group. There were significant differences in protein expression among the control group, 2-week group, 4-week group and 8-week group (Fig. 3A). ADAMTS5 began to increase at 2 weeks after modelling and maintained its expression level at 8 weeks (Fig. 3B). There was no significant difference in MMP13 expression between the control group and the 2-week model group. Protein expression was significantly different from that of the 2-week group and the 8-week group (Fig. 3C). The catabolic enzymes ADAMTS5 and MMP13 contributed to condylar cartilage destruction.

### Chondrocyte apoptosis in the OD rat models

**Fig. 4:**
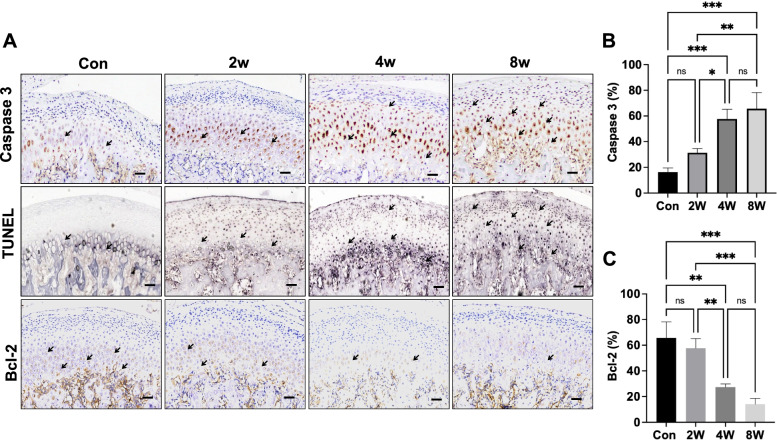
The expression of Caspase3 in the condylar cartilage of the rat models. Immunohistochemical analysis and TUNEL assay of apoptosis in mandibular condylar cartilage (**A**). Positive cells is indicted by an “** → **”. Caspase3- (**B**) cells, Bcl-2- (**C**)-positive cells were counted in the cartilage layer. Scale bars: 50 μm. **P < 0.01, ***P < 0.005. *N =* 6–8

Immunohistochemical staining for apoptosis-related markers in condylar cartilage was performed (Fig. 4A). Occlusion disorder induced an increase in Caspase3 in the OD group at 2 weeks, 4 weeks and 8 weeks (Fig. 4A). The 4-week and 8-week groups had significant increase in cartilage among the OD groups (Fig. 4B). After malocclusion stress, the 4-week and 8-week groups had higher levels of TUNEL staining positive cells in the condylar cartilage layer (Fig. 4A). The 8-week group had the significant decrease of Bcl-2 among the OD groups (Fig. 4C).

### The expression of HIF1α and HIF2α in OD rat models

**Fig. 5:**
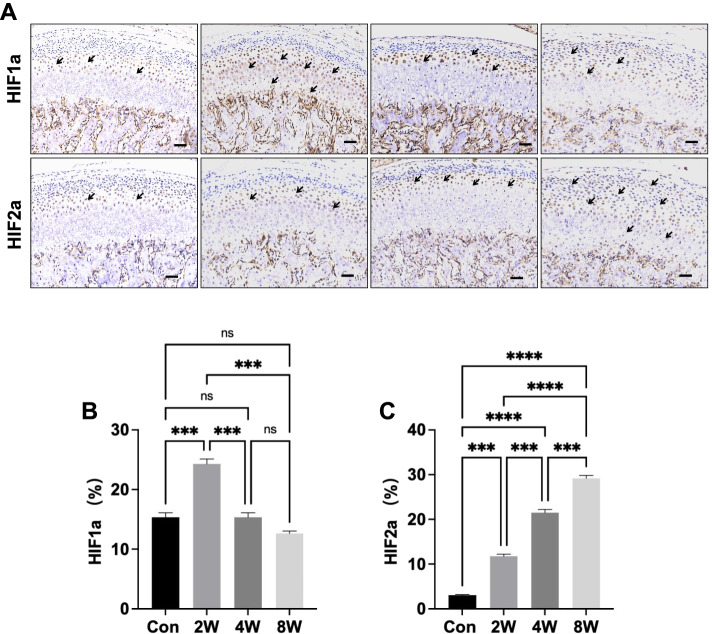
The expression of HIF1α and HIF2α in condylar cartilage in the condylar cartilage of the rat models. Immunohistochemical analysis of HIF1α and HIF2α in mandibular condylar cartilage (**A**). Positive cells is indicted by an “** → **”. HIF1α- (**B**) and HIF2α-positive (**C**) cells were counted in the cartilage layer. Scale bars: 50 μm. ***P < 0.005, ****P < 0.001. *N =* 7–8

Condylar cartilage is maintained in a low oxygen environment throughout life. Chondrocytes are therefore adapted to these hypoxic conditions. Thus, we used immunohistochemical staining to examine the expression of HIF1α and HIF2α in the condylar chondrocyte of different OD groups (Fig. 5A). Malocclusion stress significantly increased the expression of HIF1α at 2 weeks, while that of HIF1α was obviously decreased at 8 weeks compared with that at 2 weeks (Fig. 5B). HIF2α, which is a catabolic factor in chondrocytes, began to increase in the 2-week group and reached its highest expression level in the 8-week group (Fig. 5C).

## Discussion

The hallmark of OA is cartilage destruction, several factors such as catabolic enzymes and chondrocyte death include apoptosis and/or autophagy are considered for the pathogenesis [19]. Mechanical stress is considered for the pathogenesis of disruption of cartilage homeostasis and initiation of the catabolic pathway in OA [20]. The condylar cartilage of TMJ surface is covered by fibrocartilage, which is stress-sensitive [21]. Cartilage degradation is a key factor that induces excessive mechanical stress [22]. TMJOA rat models were established by unilateral molar occlusal elevation. This method is reproducible and can simulate the pathogenesis of TMJ disease [23]. Excessive mechanical loading on the normal condylar cartilage initiates the disruption of cartilage [24]. In this study, we used malocclusion stress to induce TMJOA development. We observed that the condylar cartilage structure changed after 2 weeks of malocclusion stress. Condylar cartilage degradation was obvious in the 8-week group according to Mankin and OARSI scores. The phenotype of cartilage degradation in the OD group was an early lesion of TMJOA, which was defined by Mankin scores OARSI scores and histological staining. Animal models are a critical tool to investigate the pathogenesis of TMJOA [25]. Therefore, rat OD models are suitable for investigating the disruption of cartilage matrix homeostasis and related pathogenic factors.

Unlike most hyaline articular cartilage in the appendicular joints, TMJ cartilage is classified as fibrocartilage [26]. Condylar fibrocartilage is histologically composed of the force-absorbent, proteoglycan-rich nonmineralized portion and the rigid mineralized region that abuts the subchondral bone [27]. With OA onset, chondrocytes undergo multiple changes in states including proliferation, viability and secretory profiles [28]. Acan is the major proteoglycan in articular cartilage, and the loss of Acan is a known characteristic of early OA [29]. Decreased Acan expression induced by cartilage aggrecanases and MMPs is often evident in OA cartilage [30]. ADAMTS5 is the principal aggrecanase found in animal and human OA articular cartilage [31]. The catabolic protease MMP13 contributes to OA development [32]. MMP13 is an interstitial Col II enzyme that has a particular relevance to the degradation of articular cartilage [33]. In our study, abnormal occlusion decreased the expression of Acan and Col II and enhanced the expression levels of MMP13 and ADAMTS5 in the cartilage layer of the OD group. This result suggested that malocclusion stress in turn leads to activation of biochemical pathways in chondrocytes that synthesizes cartilage-specific ECM components as well as various catabolic and anabolic factors.

Multiple factors contribute to the degradation of cartilage in OA, by either directly or indirectly regulating the anabolic and catabolic pathways of the cartilage matrix. Cartilage breakdown in OA is related not only to ECM degeneration but also to chondrocyte death. Chondrocyte death in cartilage may also occur by combination of apoptosis and autophagy depending on the stage of degenerative cartilage. Apoptosis is a highly-regulated, active process of cell death involved in development, and homeostasis of various tissues. An increase in the rate of apoptosis in articular cartilage could play an important role in OA pathogenesis. Apoptosis clearly occurs in OA cartilage and subsequently disrupts cartilage homeostasis [34]. Occlusion disorder induced high Caspase 3 expression and lower level of Bcl-2 in chondrocytes, which indicated that chondrocyte apoptosis occurred in cartilage in TMJOA. Caspase 3, the main executioner of apoptosis, is activated by intrinsic and extrinsic apoptotic pathways [35]. In addition to, anti-apoptotic members Bcl-2 family control mitochondrial apoptotic signaling by modulating mitochondrial membrane permeability [36]. Mitochondria play a key role in cellular function and survival, and oxidative stress and disrupted mitochondrial respiration were reported to promote cell death and degeneration [37].

The TMJ cartilage is maintained in a low oxygen environment throughout life [38]. Adaptation to this avascular environment is mediated by hypoxia-inducible factors, HIF1α and 2α [39]. HIF1α is expressed in human normal and OA articular chondrocytes [40]. HIF1α may be important for articular cartilage homeostasis and protective against articular cartilage degradation [41]. It suggested that HIF1α contributed to apoptosis in hypertrophic growth plate chondrocytes by liberating proapoptotic factors from blood vessels [42]. HIF2α is highly expressed in OA cartilage, which regarded as important stimuli of OA development [43]. HIF2α enhances the promoter activities of type X collagen (Col X) and MMP13 [44]. Moreover, HIF2α ectopic expression triggered articular cartilage destruction in mice and rabbits [45]. HIF1α and HIF2α play specific roles in the cellular response to a lack of oxygen, effectively regulating gene expression in response to disturbances in oxygen homeostasis. HIF2α is a potent regulator of autophagy in maturing chondrocytes and probably acts as a brake on the autophagy-accelerator function of HIF1α [46]. Our study suggested that abnormal occlusion stress aggravated the hypoxic environment in cartilage, and HIF1α increased its reactivity due to impaired TMJ cartilage damage. However, the TMJ continues to bear occlusion stress, HIF2α but not HIF1α may be essential for oxygen homeostasis in the TMJ.

## Conclusion

Our results suggested that the expression of HIF1α and HIF2α in the TMJ maybe a vital stimulatory of cartilage degeneration. HIF2α may provide a negative feedback mechanism for HIF1α during OA development. However, further studies are needed to elucidate the molecular mechanisms between HIF1α and HIF2α in OA in vitro.

## Supplementary Information

**Additional file 1: Supplemental data.** IHC negative control images with isotype-matched immunoglobulin control in sagittal sections of the TMJ mandibular condylar cartilage layers. Scale bars: 50 μm. *N =* 4.

## Data Availability

The datasets generated and/or analysed during the current study are available in the [figShare] repository, [https://doi.org/10.6084/m9.figshare.19358807.v1].

## Abbreviations

HIF1α/2α: Hypoxia-inducible factor1α/2α
OA: Osteoarthritis
OD: Occlusion disorder
TMJ: Temporomandibular joint
Col I/II/X: The type collagen I/II/X
MMP13: Matrix metallopeptidase 13
Acan: Aggrecan
Bcl-2: B-cell lymphoma-2
ECM: Extracellular matrix
ADAMTS5: Recombinant a disintegrin and metalloproteinase with thrombospondin 5
