# Supplementary material for: Hypoxia-inducible factor expression is related to apoptosis and cartilage degradation in temporomandibular joint osteoarthritis
Source: BMC Musculoskelet Disord. 2022 Jun 16;23:583. doi: 10.1186/s12891-022-05544-x (PMC9202126; doi:10.1186/s12891-022-05544-x)
Supplement: Supplementary file 1 — Additional file 1: Supplemental data. IHC negative control images with isotype-matched immunoglobulin control in sagittal sections of the TMJ mandibular condylar cartilage layers. Scale bars: 50 μm. N = 4. [file 12891_2022_5544_MOESM1_ESM.pdf]

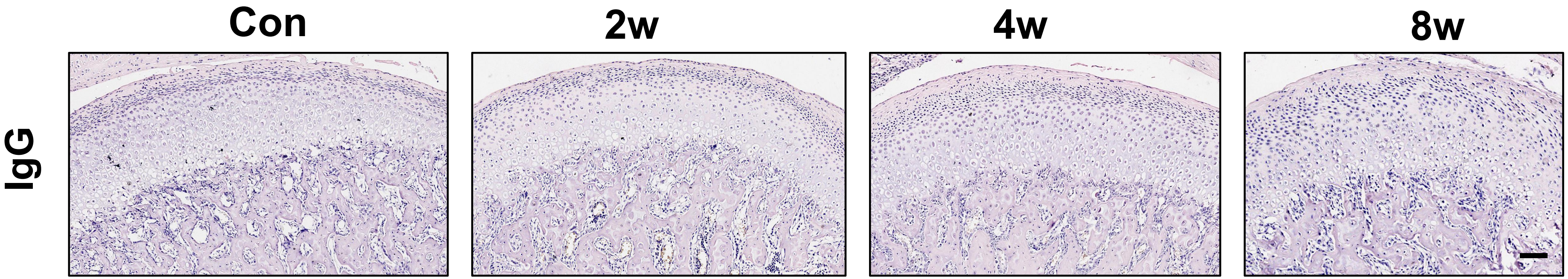

Supplemental data: IHC negative control images with isotype-matched immunoglobulin control in sagittal sections of the TMJ mandibular condylar cartilage layers. Scale bars: 50 μm. N=4.
